# Terrestrial slugs (Gastropoda, Pulmonata) in the NATURA 2000 areas of Cyprus island

**DOI:** 10.3897/zookeys.174.2474

**Published:** 2012-03-09

**Authors:** Katerina Vardinoyannis, Simon Demetropoulos, Moissis Mylonas, Kostas A.Triantis, Christodoulos Makris, Gabriel Georgiou, Andrzej Wiktor, Andreas Demetropoulos

**Affiliations:** 1 Natural History Museum of Crete, University of Crete, 71409 Herakleio Crete, Greece; 2 Cyprus Wildlife Society, P.O.Box 24281, Lefkosia 1703, Cyprus; 3 Department of Biology, University of Crete, 71409 Herakleio Crete, Greece; 4 Natural History Museum of Crete, University of Crete, 71409 Herakleio Crete, Greece; 5 21 Ethnikis Antistaseos, 3022 Limassol, Cyprus; 6 Museum of Natural History, Wrocław University, Sienkiewicza 21, 50-335 Wrocław, Poland; 7 Cyprus Wildlife Society, P.O.Box 24281, Lefkosia 1703, Cyprus

**Keywords:** Agriolimacidae, Limacidae, Milacidae, Distribution, East Mediterranean

## Abstract

Terrestrial slugs of the Island of Cyprus were recently studied in the framework of a study of the whole terrestrial malacofauna of the island. The present work was carried out in the Natura 2000 conservation areas of the island in 155 sampling sites over three years (2004–2007). Museum collections as well as literature references were included. In total six species are present in the Natura 2000 areas of the island, belonging to three families: Limacidae, Agriolimacidae and Milacidae. One of the species, *Milax riedeli*, is a new record for the island. The distribution of the species across the island and in the surrounding areas is discussed.

## Introduction

Cyprus, the largest island in the Eastern Mediterranean, has an area of 9,251 km^2^, and there are 33 special areas under nature conservation, that cover 22** **% of the total area. The designation of these areas was based mainly on habitat types, geology, knowledge of plant and vertebrate species, and on published data on invertebrates. This is in accordance with what Dimitrakopoulos et al. (2004) claim *“… it is questionable whether the inclusion of species and habitats in the list of biodiversity components of ‘community interest’ has been based on a previous detailed evaluation of regional biodiversity patterns, but rather the selection was based on the inclusion of pre-existing national ad hoc schemes”.*

Our previous knowledge of the slugs of Cyprus was mainly based on a fairly recent paper by Rähle (1991). In most other relevant papers slugs of Cyprus appear in passing, either because the work deals mainly with the neighbouring areas of the Mediterranean (Schütt 2005, Heller 2009) or they concern a particular slug taxon (Hesse 1926). Six slug species had been recorded from the island, but only three of them within a NATURA 2000 conservation area; namely *Deroceras berytensis, D. chrysorroyatissensis* and *Limax flavus* (Rähle 1984, 1991; Wiktor 2001). These slugs are recorded from six conservation areas, which are among the most popular and most visited parts of the island.

In this work we present new distributional data about the slugs of Cyprus and comments on their taxonomy when necessary. Additionally, we discuss their presence in the conservation areas and the whole island and compare it with occurrence in surrounding countries.

## Material and methods

**Map 1.**
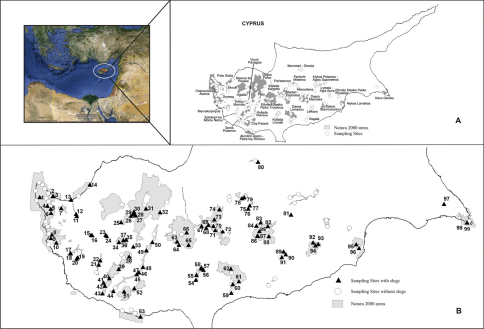
Natura 2000 areas **A** and sampling sites **B** in each area. Numbers depict sampling sites where slugs were found (their name is given in Table 1)

During the years 2004–2007 we collected land snails in all NATURA 2000 areas. Slugs were found at 99 sites within 28 of the 33 areas although we collected land snails at 155 sampling sites (Map 1, Table 1). Sampling sites were intended to cover the whole diversity (habitat and substrate) of each NATURA 2000 area. Snails and slugs were collected only during the wet period (October-April) by A. Demetropoulos (AD), S. Demetropoulos (SD), Chr. Makris (M), Chr. Makris & L. Georgiou (MG), M. Mylonas (MM), K.A. Triantis, and K. Vardinoyannis (V). We also included material from the mollusc collection of the Natural History Museum of Crete (Map 1, Table 1). After sampling, specimens were relaxed and then preserved in 75** **% ethanol. Their identification was based on anatomy of the genitalia. The material is kept in the Natural History Museum of Crete and in the Museum of Natural History, Wrocław University.

**Table 1.** Sampling sites in each NATURA 2000 area, the date of collection, the vegetation type and the dominant plant species, the substrate and the corresponding number on Map 1.

**NATURA 2000 area**	**Sampling site with slugs**	**Date**	**Number on map**	**Dominant Vegetation**	**Substrate**
Agiatis	Agiatis–Agia S (CY411–1)	14/3/2006	25	Pine forest (*Pinus brutia*)	Diabase dykes
	Agiatis–500m from Tarmac CY411–2	14/3/2006	26		
Akrotirio Aspro–Petra tou Romiou	Aspro cape–Petra tou Romiou (eastern valley)	29/1/2005	53	Phrygana (*Sarcopoterium spinosum*; Maquis (*Olea europaea*, *Ceratonia siliqua*)	Biocalcarenites, sandstones
Alykes Larnakas	Alyki, Tekes (site 1)	15/1/2005	95	Plantations with *Acacia*	Sand, silts
	Larnaka salt marsh new buildings	19/11/2005	96		
Alykos Potamos–Agios Sozomenos	Kotsiatis Dam (CY202–1)	4/3/2006	81	Phrygana (*Sarcopoterium spinosum*)	Pillow lavas, Olivine - Pyroxene
Cha Potami	Cha river, Kato Archimantria	5/3/2005	52	Maquis (*Olea europaea* & *Ceratonia siliqua*)	Chalks, marls
	Cha river, Orites	5/3/2005	51		
Chersonisos Akama	Agios Kononas springs	24/11/2005	1	Maquis (*Juniperus phoenicea*, *Olea europaeaCeratonia siliqua*); Phrygana (*Sarcopoterium spinosum*); Pine forests (*Pinus brutia*)	Limestone
	Agios Minas spring CY410–11	15/3/2005	4		
	Akamas (may be Loutra tis Afroditis)	1/10/1989	3		
	Akamas forest CY 410–8	12/3/2005	6		
	Akamas, Loutra tis Afroditis CY 410–10	15/3/2005	2		
	Avakas	19/2/2005	8		
	Mountiko maquis CY410–3	24/1/2005	9		
	Petratis gorge	23/11/2005	7		
	Pittokopos CY410–13	16/3/2005	5		
	Pykni forest CY410–4	15/2/2005	10		
Dasos Lemesou–Periochi Kyparisia	Germasogeia north, at the stream (CY 20)	20/2/2000	59	Pine forest (*Pinus brutia*); Maquis (*Olea europaea*, *Ceratonia siliqua*)	Serpentinized harzburgites, Diabase dykes and gabbros
	Lemesos forest, Akrounta river valley	11/2/2006	62		
	Lemesos forest, Foinikaria	2/1/2005	61		
	Lemesos forest, Germasogeia dam, Foinikaria	2/1/2005	60		
Dasos Machaira	Gionia Camp (CY204–2)	17/3/2006	87	Forest (*Quercus alnifolia*, *Pinus brutia*)	Diabase dykes and pillow lavas
	Gionia Valley north (CY204–3)	17/3/2006	85		
	Kapedes (CY204–1)	17/3/2006	82		
	Kiona ~1380m alt	22/12/2006	86		
	Lazania CY 204–7	27/12/2006	84		
	Machairas, Kyprovasa– Arkatzi tou Klosmatou	22/12/2006	88		
	Philani Pine CY 204–6	27/12/2006	83		
Dasos Pafou	Dasos Pafou Tripyla	7/1/2006	29	Forests with Pines (*Pinus brutia*) and Cedars (*Cedrus brevifolia*)	Diabase dykes
	Gerakies (CY 206–3)	15/3/2006	32		
	Kedron valley	13/3/2005	28		
	Kedron valley (CY 10)	18/2/2000	27		
	Kremnos tis Pellis CY 206–1	15/3/2006	30		
	Roudia Bridge (site CY7)	17/2/2000	33		
	Sylladin tou Petrou near Tsakistra	15/3/2006	31		
Dasos Stavrovouniou	Stavrovouni, entrance of monastery	19/12/2006	93	Pine forest (*Pinus brutia*); Maquis (*Olea europaea*, *Ceratonia siliqua*)	Diabase dykes and pillow lavas
	Stavrovouni, low	19/12/2006	92		
	Stavrovouni, NE low	19/12/2006	94		
Drymou	Drymou Oak	28/3/2007	16	Riparian	Biocalcarenites, sandstones
	Drymou Valley	28/3/2007	15		
Episkopi tou Morou Nerou	Ezousas Alder (CY405–1)	11/2/2006	22	Maquis (*Quercus coccifera*)	Sand, silts, clays
	Ezousas Pseudogarrigue (CY405–2)	11/2/2006	21		
Ethniko Dasiko Parko Troodous	Caledonian falls	22/11/2005	63	Pine forests (*Pinus nigra*, *Pinus brutia*)	Gabbros, harzburgites
	Mesapotamos waterfall	22/11/2005	65		
	Platres to Mesapotamos	22/11/2005	64		
	Troodos mt., Almyrolivado	26/11/2005	66		
Kavo Gkreko	Cavo Gkreko Rocks	9/1/2005	99	Maquis (*Juniperus phoenicea*); Phrygana (*Sarcopoterium spinosum*)	Limestone
	Gkreco cape at Agioi Anargyroi (CY 24)	21/2/2000	98		
	Paralimniou lake	9/1/2005	97		
Koilada Diarizou	Diarizos Arminou	28/1/2006	49	Maquis (*Olea europaea*, *Ceratonia siliqua*); Phrygana (*Sarcopoterium spinosum*); Pine forests (*Pinus brutia*)	Chalks, sand, lava breccia
	Diarizos gorge, after Kikisia (CY 8)	17/2/2000	47		
	Diarizos Kidasi	22/1/2006	45		
	Diarizos Nikokleia	28/1/2006	44		
	Diarizos valley, Gefyri	22/1/2005	48		
	Diarizos valley, Petres ton Hasanpoulion	22/1/2005	46		
Koilada Limnati	Limnatis valley, 1 km west of the bridge, 400 m alt.	5/12/2004	58	Maquis (*Olea europaea*, *Ceratonia siliqua*)	Chalks, serpentinite
	Limnatis valley, Alassa	19/11/2005	55		
	Limnatis valley, Mantra	18/2/2006	54		
	Palia Korfi, approx. 500 m alt., Limnatis valley	4/12/2004	56		
	Palia Korfi, river below at Limnatis bridge	4/12/2004	57		
Kritou Marotou	Kritou Marotou Cultivations	27/3/2007	24	Riparian and cultivations	Chalks, marls, clays
	Kritou Marotou Oak	27/3/2007	23		
Lefkaron	Lefkara–Agios Minas	25/2/2006	91	Presteppe scrub (*Genista fasselata*; Maquis (*Quercus coccifera*)	Chalks, marls and pillow lavas
	Lefkara 600 m alt	19/11/2005	89		
	Lefkara croosroad to Kato Drys	11/3/2006	90		
Madari–Papoutsa	Kyperounda (CY205–1)	25/3/2006	67	Forest (*Quercus alnifolia*, *Pinus brutia*)	Diabase dykes and pillow lavas
	Lagoudera valley	20/12/2006	70		
	Lagoudera, 6 km north	21/12/2006	73		
	Papoutsa ~1240m alt.	20/12/2006	72		
	Pitsilia district, Kyperounta	15/4/2001	68		
	Polystypos Fountoukies	17/12/2006	71		
	Spilia–Madari, 1250m alt.	17/12/2006	69		
	Xyliati dam, low	21/12/2006	74		
Mammari–Deneia	Mammari 1st site	21/11/2005	80	Phrygana (*Sarcopoterium spinosum*)	Biocalcarenites, sandstones
Maroullena	Maroulena gorge	17/12/2006	75	Pine forest (*Pinus brutia*) and Riparian	Pillow lavas
	Maroulena’s Dam	23/12/2006	76		
	Maroulena’s Pine	23/12/2006	77		
Mavrokolympos	Agios Neophytos Valley CY408–4	13/1/2006	20	Maquis (*Olea europaea*, *Ceratonia siliqua*); Phrygana (*Sarcopoterium spinosum*)	Chalks, marls
	Agios Neophytos Garrigue CY408–3	13/1/2006	19		
	Mavrokolymbos Garrigue CY408–1	9/1/2006	18		
	Mavrokolymbos Stream CY408–2	9/1/2006	17		
Periochi Mitserou	Mitsero–Agios Panteleimonas	11/3/2006	79	Phrygana (*Sarcopoterium spinosum*); Pine forest (*Pinus brutia*)	Chalk & Limestone
	Mitsero Pinewood & valley	11/3/2006	78		
Platy	Platy area, crossroad Kelefos–Kaminaria–Milikouri	19/2/2005	50	Pine forest (*Pinus brutia*)	Diabase dykes
Polis Gialia	Gialia Acacia CY401–1	8/1/2006	14	Plantations with *Acacia*	Calcarenites, sands, gravel
Skoulli	Chrysochou River CY409–2	15/1/2006	12	Woodland (*Quercus infectoria*); Riparian (*Platanus orientalis*, *Nerium oleander*)	Sand, silts, gravel
	Goudi Oak CY409–1	15/1/2006	11		
	Polis Camp river CY409–3	15/1/2006	13		
Vouni Panagias	Agia Moni south of Panagia	22/12/2004	34	Forests (*Quercus infectoria*, *Pinus brutia*)	Chalks, marls
	Makries Limnes Chasanpoulion–Eryfiou–Profitis Ilias	23/12/2004	36		
	Profitis Ilias	1/2/2005	35		
	Vloudkia	23/12/2004	37		
Xeros Potamos	Asprokremnos Pools CY407–1	2/2/2006	43	Phrygana (*Sarcopoterium spinosum*); Maquis (*Olea europaea*, *Ceratonia siliqua*); Pine forests (*Pinus brutia*)	Chalks, marls
	Finikas CY407–2	2/2/2006	42		
	Nata Pine CY407–5	6/2/2006	39		
	Xeros Army CY407–3	2/2/2006	41		
	Xeros Rock CY407–4a	3/2/2006	38		
	Xirou valley, Nata	5/2/2005	40		
Stavros tis Psokas	Agios Merkourios			Pine forests	Diabase dykes
	Stavros tis Psokas (CY 11)	18/2/2000			
Ethniko Dasiko Parko Rizoelias	No slugs were found in these areas				
Asgata					
Lympion–Agia Anna					
Peristerona					
Koilada Kargotis					

In Table 1 we give the sampling sites in each NATURA 2000 area, the date of collection, the vegetation type, the dominant plant and the substrate.

Initially we assembled all data from the literature, including the doubtful names. These are presented and discussed separately for each species.

## Results

In total we found six slug species belonging to three families - Agriolimacidae, Limacidae and Milacidae. Below we present analytically for each species the collecting sites in each NATURA 2000 conservation area (bold), data from literature, and if necessary comments on its systematics.

### Family Agriolimacidae Wagner, 1935

***Deroceras berytensis* (Bourguignat,1852)**

This species had been reported from Akrotirio Aspro–Petra tou Romiou; Alykes Larnakas; Chersonisos Akama; Ethniko Dasiko Parko Troodous; Koilada Diarizou; Polis Gialia and Vouni Panagias (Rähle 1984, 1991).

**Figure 1.**
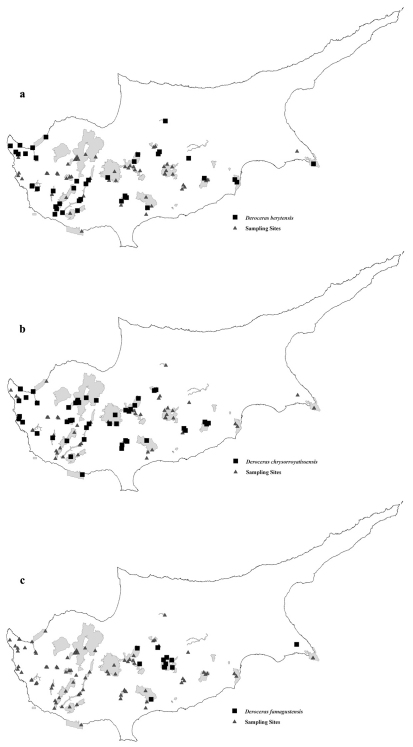
Distribution of **a** *Deroceras berytensis* **b** *Deroceras chrysorroyatissensis* and **c** *Deroceras famagustensis* in the Natura 2000 areas of conservation in Cyprus Island.

We found it in (Fig. 1a):**Alykes Larnakas:** Larnaka salt marsh new buldings, 19.11.05, M; Alyki Tekes, 15.1.05, MG. **Alykos Potamos – Agios Sozomenos:** Kotsiatis Dam (CY202–1), 4.3.2006, SD. **Cha Potami:** Cha river, Kato Archimandria, 5.03.05, MG. **Chersonisos Akama:** Agios Minas spring CY 410–11, 15.3.05, AD; Agios Kononas springs, 24/11/2005, AD; Akamas (may be Loutra tis Afroditis), 1.10.1989, MM; Petratis gorge, 23.11.05, MM; Pittokopos CY 410–13, 16.03.05, SD. **Dasos Lemesou – Periochi Kyparisia:** Lemesos forest, Germasogeia dam, Foinikaria, 2.1.05 MG. **Dasos Pafou:** Roudia bridge (CY7), 17.2.00, MM. **Dasos Stavrovouniou:** Stavrovouni, low, 19.12.06 MM. **Episkopi tou Morou Nerou:** Ezousas Alder (CY405–1), 11.02.06, SD. **Kavo Gkreko:** Cavo Greko Rocks, 9.1.05, MM; Gkreko cape at Agioi Anagyroi (CY24), 21.02.00, MM. **Koilada Diarizou:** Diarizou gorge, after Kikisia (CY 8), 17.02.00, MM; Diarizos Arminou, 28.1.06, MG; Diarisos Kidasi, 22.1.06, AD; Diarizos Nikokleia, 28.1.06, MG; Diarizos valley, Petres ton Hasanpoulion, 22.1.2005, MG. **Koilada Limnati:** Limnatis valley, Alassa, 19.11.05, MG; Palia Korfi, river below at Limnatis bridge, 4.12.04, MM; Palia Korfi, approx. 500 m alt. Limnatis valley, 4.12.04, MM; Limnatis valley, 1 km west of the bridge, 400 m alt., 4/12/2004, MM. **Madari – Papoutsa:** Lagoudera, 6 km north, 21.12.06, V; Xyliati dam, low, 21.12.06, MM. **Mammari – Deneia:** Mammari 1st site, 21.11.2005, V. **Maroullena:** Maroulena’s Dam, 23.12.06, SD; Maroulena gorge, 17.12.06, MM, V, SD. **Mavrokolympos:** Mavrokolymbos stream CY408–2, 9.1.06, SD; Mavrokolymbos Garrigue CY408–1, 9.01.06 SD; Agios Neophytos valley (CY 408–4), 13.1.06 SD. **Polis Gialia:** Gialia Acacia CY401–1, 8.01.06, AD & SD. **Skoulli:** Chrysochou River CY 409–2, 15.01.06, SD; Polis Camp river CY409–3, 15.01.06, SD. **Xeros Potamos:** Asprokremnos Pools CY407–1, 2.2.06, AD; Xirou valley Nata, 5.2.05 MG; Finikas CY407–2, 2.02.06, AD & SD; Xeros Army (CY 407–3), 2.2.06, AD & SD; Xeros Rock CY407–4a, 3.02.06, AD & SD

**Comments on systematic characters**

The colour of alcohol-preserved specimens ranges from cream to dark brown, especially on the back and mantle; the dark pigment is distributed more or less evenly with no distinct spots; when spots are present, they are blurred and their margins fuse (they are never black dots). These external characters are always combined with the following anatomical characters: long caecum, forked penial gland with a distinct common stalk, more or less half as long as the entire gland. The two branches of the penial gland are covered by glandular papillae. The stimulator in the penis is wide, flat, and bowl-shaped.

The species varies very widely (Wiktor 2000); a few very similar slugs, of unclear taxonomic status, are found in the literature. *Agriolimax cyprius* Simroth, 1906 was described from Cyprus as two forms (*Agriolimax cyprius* and *Agriolimax cyprius coeciger* Simroth, 1906). The description, however, is so laconic that it is impossible to say which slugs Simroth (1906) was dealing with (the types most probably have not been preserved). Rähle (1984) commented on these slugs saying that the slugs from Cyprus differed in a few anatomical details from *Deroceras berytensis* earlier described from Lebanon. The slugs from Cyprus are often smaller; differ somewhat in the appearance of their glandula hermaphroditica, caecum length, penial gland and stimulator. Rähle (1984) suspected that it might be only a form of the variable *Deroceras berytensis* In our opinion these comments are justified and agree with our own observations; according to Wiktor (2000), at the current state of knowledge it is reasonable to regard the name *Agriolimax cyprium* as a junior synonym of the widely distributed and very variable *Deroceras berytensis*rather than use names without knowing what they refer to. It cannot be excluded that Simroth (1906) was dealing not only with *Deroceras berytensis* but also for example with *Deroceras famagustensis*.

***Deroceras chrysorroyatissensis* Rähle, 1984**

This species had been reported from Akrotirio Aspro–Petra tou Romiou and Vouni Panagias (Rähle 1991).

We found it in (Fig. 1b): **Agiatis:** Agiatis–Agia S (CY411–1), 14.03.06, MM, V, AD, SD; Agiatis – 500m from Tarmac CY411–2, 14.03.06, MM, V, AD, SD. **Akrotirio Aspro – Petra tou Romiou:** Aspro cape – Petra tou Romiou (eastern valley), 29.01.05, M. **Cha Potami:** Cha river Orites, 5.03.05, MG. **Chersonisos Akama:** Pykni forest CY 410–4, 15.02.05, SD ? juv.; Akamas (may be Loutra tis Afroditis), 1.10.89, MM; Akamas, Loutra tis Afroditis CY410–10, 15.03.05, SD; Avakas, 19.02.05, SD; Mountiko maquis CY410–3, 24.01.05, SD ? juv.; Petratis gorge, 23.11.05, MM; Akamas forest CY 410–8, 12.03.05, SD. **Dasos Lemesou – Periochi Kyparisia:** Lemesos forest, Akrounta river valley, 2.01.06, MG. **Dasos Pafou:** Gerakies CY206–3, 15.03.06, MM, V, AD, SD; Kremnos tis Pellis CY 206–1, 15.3.06. MM, V, AD, SD; Sylladin tou Petrou CY206–2, 15.03.06, MM, V, AD, SD; Kedron valley, 13.03.05, MG; Dasos Pafou–Tripyla, 7.01.06, AD; Kedron valley (CY 10), 18.02.00, V. **Dasos Stavrovouniou:** Stavrovouni, entrance of monastery, 19.12.06, MM, V; Stavrovouni, NE low, 19.12.06, MM, V; Stavrovouni, low, 19.12.06, MM, V. **Episkopi tou Morou Nerou:** Ezousas Pseudogarrigue (CY 405–2), 11.02.06, SD. **Ethniko Dasiko Parko Troodous:** Mesapotamos waterfall, 22.11.05, V; Troodos mt. Almyrolivado, 26.11.05, M; Platres to Mesapotamos, 22.11.05, MM. **Koilada Diarizou:** Diarizos Arminou, 28.01.06, MG; Diarizou valley, Gefyri, 22.1.05, MG. **Koilada Limnati:** Limnatis valley, Alassa, 19.11.05, MG; Palia Korfi, river below at Limnatis bridge, 5.12.04, MG; Palia Korfi, approx. 500m alt. Limnatis valley, 5.12.04, MM; Limnatis valley, Mantra, 18.02.06, M. **Lefkaron:** Lefkara 600m alt., 19.11.05, MM. ? juv.; Lefkara croosroad to Kato Drys, 11.03.06, MM. **Madari – Papoutsa**: Xyliati dam, low, 21.12.06. MM, V; Lagoudera 6 km north, 21.12.06, MM, V; Pitsilia district, Kyperounta, 15.04.01 ? juv.; Kyperounta (CY 205–1), 25.03.06, SD; Spilia – Madari, 1250m alt., 17.12.06, MM, V. **Mavrokolympos:** Agios Neophytos Garrigue CY 408–3, 13.01.06, SD. **Periochi Mitserou:** Mitsero, Pinewood & valley CY203–2, MM, V, AD, SD; Mitsero–Agios Panteleimonas, 11.03.06, MM, V, AD, SD. **Platy:** Platy area crossroad Kelefos–Kaminaria– Milikouri, 19.02.05, MG. **Skoulli:** Polis Camp river CY409–3, 15.01.06, SD; Goudi Oak CY409–1, 15.01.06, AD. **Vouni Panagias**: Makries Limnes Chasanpoulion – Eryfiou – Profitis Ilias, 23.12.04, V; Profitis Ilias, 1.02.05, MG; Vloudkia, 23.12.04, MG; Agia Moni south of Panagia, 22.12.04, MG. **Xeros Potamos:** Nata Pine CY407–5, 6.02.06, SD.

**Comments on systematic characters**

The slug is easy to recognise even based solely on its external appearance. As emphasised by Rähle (1984), the species is characterised by very little variation of the characters which are regarded as diagnostic: the external colour pattern on the body, the penis shape, with its external and internal accessory structures, and the absence of a rectal caecum. Only the appendix at the posterior end of the penis may vary in shape. This constancy of characters is exceptional within the genus *Deroceras* In all likelihood it is endemic to Cyprus. Otherwise, a slug with such a characteristic appearance would have been noticed elsewhere.

***Deroceras famagustensis* Rähle, 1991**

It had not been reported from any NATURA 2000 site.

We found it in (Fig. 1c): **Dasos Lemesou – Periochi Kyparisia:** Lemesos forest, Foinikaria, 2.1.2005, MG. **Dasos Machaira:** Gionia Valley north (CY204–3), 17.03.06, SD; Lazania CY 204–7, 27.12.06, SD; Kapedes (CY204–1), 17.03.06, MM; Kiona – 1380 m alt., 22.12.06, MM, V; Machairas Kyprovasa–Arkatzi tou Klosmatou, 22.12.06, MM, V; Gionia Camp CY204–2, 17.03.06, SD; Philani Pine CY204–6, 27.12.06, SD. **Kavo Gkreko:** Paralimniou lake, 9.1.2005, MG. **Madari – Papoutsa:** Papoutsa 1240 m alt., 20.12.06, MM; Xyliati dam, low, 21.12.06, MM, V. **Maroullena:** Maroulena’s Pine, 23.12.06, D.

**Comments on systematic characters**

We found only unspotted specimens, which is in agreement with Rähle (1991), though the mantle often gives an impression of being speckled with a dark pigment. Thus the colour is not uniform. The back, outside the mantle, is covered by a pattern in the form of a dark reticulation following the system of skin grooves; the dark pigment concentrates in these grooves. Most specimens have thin and soft skin. The penis is thin-walled, of varying shape. Inside it, complicated structures adhering to the penis wall form a kind of pocket. When everted, they form a nearly circular shallow bowl or a slightly concave shield. Rähle (1991) mentions the absence of a stimulator, but apparently this structure should be regarded as one of an unusual shape. Its position within the penis, as well as when everted, clearly indicates that this is its role. The caecum is vestigial. This species is endemic to Cyprus.

There are three Natura 2000 areas, namely Drymou, Stavros tis Psokas and Kritou Marotou, where we found only juvenile *Deroceras*.

### Family Limacidae Rafinesque, 1815

***Limax flavus* Linnaeus, 1758**

This species had been reported from Akrotirio Aspro – Petra tou Romiou and Ethniko Dasiko Parko Troodous (Rähle 1991).

**Figure 2.**
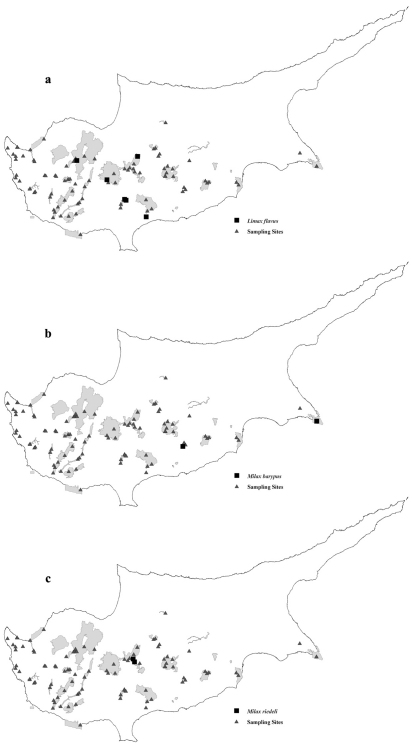
Distribution of **a** *Limax flavus* **b** *Milax barypus* and **c** *Milax riedeli* in the Natura 2000 areas of conservation in Cyprus Island.

We found it in (Fig. 2a): **Dasos Lemesou – Periochi Kyparisia:** Germasogeia north, at the stream (CY20), 20.2.00, V. **Dasos Pafou:** Kedron valley, 13.3.05, MG. **Ethniko Dasiko Parko Troodous:** Caledonian Falls, 22.11.05, MM. **Koilada Limnati:** Palia Korfi, river below at Limnatis bridge, 5.12.04, MG; Palia Korfi, approx. 500 m alt, Limnatis valley, 4.12.04, MM. **Madari – Papoutsa:** Xyliati dam, low, 21.12.06, MM.

### Family Milacidae Ellis, 1926

***Milax barypus* Bourguignat, 1866**

It had not been reported from any NATURA 2000 site.

We found it in (Fig. 2b): **Kavo Gkreko:** Gkreko cape at Agiol Anargyrol CY24, 21.02.00, MM; Cavo Gkreko, 9.01.2005, M. **Lefkaron:** Lefkara – Agios Minas, 25.02.06, MG ? juv.

This species is endemic to Cyprus.

***Milax riedeli* Wiktor, 1986**

This species had not yet been reported from the island.

We found it in (Fig. 2c): **Madari – Papoutsa:** Polystypos with hazel, 17.12.06, MM, V ? juv.; Lagoudera valley, 20.12.06, MM, V, SD.

The above list represents the current state of knowledge of slug diversity within the NATURA 2000 areas of conservation of Cyprus Island. To provide a more complete picture of the fauna we have to add that Bourguignat (1853) recorded *Limax antiquorum* A.E.Férussac,1819 and *Limax variegatus* **Férussac** [= *Limax variegatus* Draparnaud 1805] from the island, but he said that “they do not look like the true European species (naming *Limax cinereus*)”. It will be possible to clarify these names after knowing the slugs in the whole island and not only in the areas of conservation. Also Rähle (1991) listed *Deroceras cyprium* but this most probably refers to *Deroceras berytensis*.

## Discussion

In the Natura 2000 areas of conservation there are six slug species, one of them, *Milax riedeli*, a new record for the whole island. According to the literature there were only three species known from the Natura 2000 areas; thus we have doubled the number of species. *Tandonia sowerbyi* is the only species that has been recorded from the island (Rähle 1991; pers. obs.) but still not in the NATURA 2000 areas. However, we cannot exclude the possibility of finding this species in one of the areas in the future, since in the Mediterranean it can be found in undisturbed as well as disturbed places (Wiktor 2001). The slug fauna in the NATURA 2000 areas of conservation of Cyprus appears at first glance equally rich as the corresponding areas of the island of Crete (seven species on Crete, Vardinoyannis 1994, Wiktor et al. 1994). However, on Crete there are 10 slug species on the whole island, and thus only 70% of the slug fauna is found in the conservation areas, compared to 85.7% on Cyprus. It seems that the slug fauna of Cyprus is well represented in the NATURA 2000 areas, regardless on which basis these areas were proposed.

*Deroceras chrysorroyatissensis* and *Deroceras berytensis* are the most widespread species, both present in 19 areas; but the latter is distributed all around the island, while *Deroceras chrysorryatissensis* has a more restricted distribution. All the other species are found in 2–5 areas of conservation.

Three species are endemic to the island, namely *Deroceras chrysorryatissensis*, *Deroceras famagustensis* and *Milax barypus*. One species, *Milax riedeli* is distributed on Cyprus and the southeast coast of Turkey (Schütt 2005), while *Limax flavus* is found all around the Mediterranean and Europe (Wiktor 2001) and *Deroceras berytensis* all around the eastern Mediterranean (Heller 2009; Wiktor 2000; Schütt 2005).

There are five NATURA 2000 areas where no slugs were found – Ethniko Dasiko Parko Rizoelias, Asgata, Lympion – Agia Anna, Peristerona and Koilada Kargotis. In the first two areas the substrate is mainly gypsum which is most probably the reason for their absence. In the other areas the substrate is limestone, and the vegetation is Mediterranean scrubland (maquis and phrygana). Based on all the characteristics of these areas there is no obvious explanation for the absence of slugs, and we consider it as possible that slugs might be found in the future.

**Figure 3.**
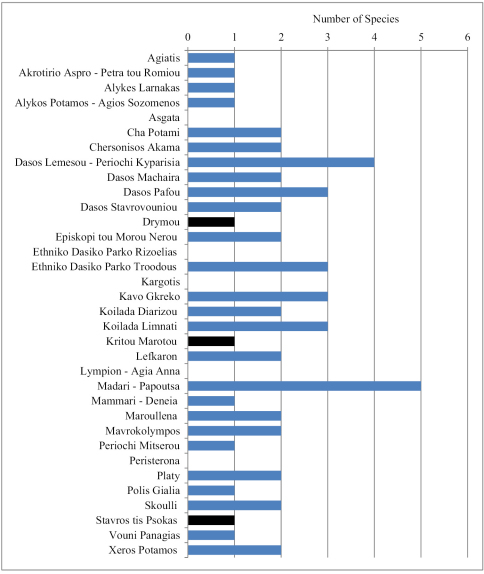
Number of slug species in each Natura 2000 area. Bars in black depict juvenile individuals.

The richest Natura 2000 area is Madari – Papoutsa, in the center of the island, with five of the six species present (Fig. 3). This is followed by Dasos Lemesou – Periochi Kyparisia. There is only one site, Xyliati dam, where all three *Deroceras* species co-occur; additionally, at this site *Limax flavus* was also found. In most other localities there is only one slug species present, usually *Deroceras berytensis*. Milacidae are very restricted: each species has been found in only two of the NATURA 2000 areas.

*Deroceras chrysorroyatissensis* was known from very few localities in the southwest part of the island (Rähle 1991; Wiktor 2001) but with our study it appears that this species has a wider distribution, it is absent from the northern and the easternmost part of Cyprus.

*Deroceras famagustensis* had been reported only from Cavo Gkreco, the southeastern peninsula of the island, but we found it in the central part of Cyprus. Further studies could enlarge its known distribution still further.

Until recently, *Limax flavus* had been known only from sites near human settlements, but we found it also in more natural areas.

*Milax barypus* was known from three suburban areas in the eastern and northern part of the island but we also found it in central Cyprus.

*Milax riedeli* is recorded for the first time from the island. It is distributed in the central part of the island in the area of Madari–Papoutsa.

The presence of the slug species does not seem to correlate with vegetation, rainfall, altitude or the substrate of the site they were found.

All slugs were active only during the wet period; *Limax flavus* in the urban areas is an exception, as it was found active also during the dry season, even in summer (pers. obs.).

In the future we will present more data on this group since we are currently studying the terrestrial malacofauna of the whole island.
